# Prevalence and determinants of asymptomatic *Leishmania* infection in HIV-infected individuals living within visceral leishmaniasis endemic areas of Bihar, India

**DOI:** 10.1371/journal.pntd.0010718

**Published:** 2022-08-30

**Authors:** Raman Mahajan, Sophie I. Owen, Shiril Kumar, Krishna Pandey, Shahwar Kazmi, Vikash Kumar, Emily R. Adams, Amit Harshana, Sakib Burza

**Affiliations:** 1 Médecins Sans Frontières, New Delhi, India; 2 Department of Tropical Disease Biology, Liverpool School of Tropical Medicine, Liverpool, United Kingdom; 3 Rajendra Memorial Research Institute of Medical Science, Patna, Bihar, India; 4 London School of Hygiene and Tropical Medicine, London, United Kingdom; The University of Kansas, UNITED STATES

## Abstract

People living with HIV (PLHIV) have an increased risk of developing visceral leishmaniasis (VL) and poor outcomes compared to HIV negative individuals. Here, we aim to establish the prevalence and determinants of asymptomatic *Leishmania* infection (ALI) in a cohort of PLHIV in Bihar, India. We hoped to evaluate optimal diagnostic algorithms to detect ALI in PLHIV. We conducted a cross-sectional survey of PLHIV ≥18 years of age with no history or current diagnosis of VL or post kala-azar dermal leishmaniasis (PKDL) at anti-retroviral therapy centres within VL endemic districts of Bihar. ALI was defined as a positive rK39 enzyme-linked immunosorbent assay (ELISA), rK39 rapid diagnostic test (RDT) and/or quantitative polymerase chain reaction (qPCR). Additionally, the urinary *Leishmania* antigen ELISA was evaluated. Determinants for ALI were established using logistic regression and agreement between diagnostic tests calculated using Cohen’s Kappa. A total of 1,296 PLHIV enrolled in HIV care, 694 (53.6%) of whom were female and a median age of 39 years (interquartile range 33–46), were included in the analysis. Baseline prevalence of ALI was 7.4% (n = 96). All 96 individuals were positive by rK39 ELISA, while 0.5% (n = 6) and 0.4% (n = 5) were positive by qPCR and rK39 RDT, respectively. Negligible or weak agreement was seen between assays. Independent risk factors for ALI were CD4 counts <100 (OR 3.1; 95% CI 1.2–7.6) and CD4 counts 100–199 (OR = 2.1;95% CI:1.1–4.0) compared to CD4 counts ≥300, and a household size ≥5 (OR = 1.9;95% CI:1.1–3.1). A total of 2.2% (n = 28) participants were positive by *Leishmania* antigen ELISA, detecting 20 additional participants to the asymptomatic cohort. Prevalence of ALI in PLHIV in VL endemic villages in Bihar was relatively high. Using the *Leishmania* antigen ELISA, prevalence increased to 9.0%. Patients with low CD4 counts and larger household size were found to have significantly higher risk of ALI.

**Trial Registration:** Clinical Trial Registration CTRI/2017/03/008120.

## Author summary

People living with HIV (PLHIV) are more likely to develop visceral leishmaniasis (VL) and are more likely to have poor outcomes associated with VL-HIV coinfection than HIV-negative individuals. While an estimated 3–14% of immunocompetent individuals living in endemic areas show serological evidence of asymptomatic infection with VL, such data on *Leishmania* infection in PLHIV in India are lacking. As such we sought to establish the prevalence and determinants of asymptomatic *Leishmania* infection (ALI) in PLHIV in VL endemic districts of Bihar, India using a combination of molecular and serological assays. We further expand our definition of ALI to include the urinary Leishmania antigen ELISA. The prevalence of ALI in a population of PLHIV residing in VL endemic districts of Bihar, India was found to be as high as 9.0% (n = 116). The majority of individuals were detected by the rK39 enzyme-linked immunosorbent assay (ELISA) (7.4%, n = 96), and very few individuals were detected by quantitative polymerase chain reaction (qPCR) (0.5%, n = 6) or the rK39 rapid diagnostic test (RDT) (0.4%, n = 5). In total, 2.2% (n = 28) were positive by urinary *Leishmania* antigen ELISA. Low CD4 counts and larger household size were found to be risk factors for ALI. Here, we add to the evidence base for *Leishmania*-HIV coinfection on the Indian subcontinent.

## Introduction

Human immunodeficiency virus (HIV) and leishmaniasis are found to be co-endemic in several regions [1]. Infection with the protozoa *Leishmania donovani* (*L*. *donovani*) can remain asymptomatic or can lead to symptomatic visceral leishmaniasis (VL), with asymptomatic infections outnumbering clinical infections by an estimated 9 times on the Indian subcontinent (ISC) [2]. The risk of developing VL in people living with HIV (PLHIV) is estimated to be far higher compared to HIV-negative individuals [3]. Furthermore, the risk of poor clinical outcomes from VL-HIV such as treatment failure and relapse are increased, with the virus and parasite mutually accelerating disease progression [3–6].

In 2019, within the state of Bihar, India, there were estimated to be 134,000 PLHIV representing 0.18% of the state population and the second-highest number of new infections behind the state of Maharashtra [7]. There are limited data on VL-HIV coinfection in India. In 2014, a consecutive HIV screening of 2,077 people over 13 years of age with VL infection in Bihar, found a 5.6% coinfection rate [8]. The Indian National Vector Borne Disease Control Programme (NVBDCP) subsequently recommended screening all patients presenting with VL for HIV, and inversely recommended screening all HIV patients living in VL endemic areas for VL [9]. However, there are no optimal screening methods for the latter cohort [10].

Furthermore, where asymptomatic *Leishmania* infection (ALI) may represent an anthroponotic reservoir on the ISC [11], data on prevalence and determinants for ALI and guidelines on optimal screening algorithms in PLHIV are absent. This evidence gap is potentially important; the majority of patients diagnosed with VL-HIV present at a late stage with advanced HIV; assuming a reasonably high progression from asymptomatic to symptomatic VL infection in PLHIV, the utility of a tool that could potentially be used to identify the subclinical form earlier could be of major benefit in the early detection and management of this co-infection. Such screen-and-treat strategies in East Africa have been conceptionally described elsewhere [12].

As the effort to eliminate VL as a public health problem has progressed substantially in the ISC, the proportion of patients with VL-HIV has increased both in absolute numbers and as a proportion of all VL cases. Indeed, a more recent analysis of the epidemiological spread and impact of VL-HIV has suggested that the presence of VL-HIV cases was associated with a greater than two-fold increase in VL incidence at the village level, with an incidence risk ratio similar to that of post kala-azar dermal leishmaniasis (PKDL) [13]. As such, establishing the scale of asymptomatic infections in HIV patients may contribute significantly to improved programmatic policy in sustaining elimination targets.

Molecular methods to detect *Leishmania* kinetoplast DNA (kDNA) such as quantitative polymerase chain reaction (qPCR) are highly sensitive techniques but require a good laboratory set-up and expertise. The rK39 enzyme-linked immunosorbent assay (ELISA) and rapid diagnostic test (RDT) detect anti-*Leishmania* antibodies and are used in the diagnosis of VL, however, they may detect convalescence and have shown a reduced sensitivity in HIV infection in a study in Ethiopia [14]. The *Leishmania* antigen ELISA detects *Leishmania* carbohydrate in a urine sample, making it a non-invasive test that detects active infection [15]; however, the assay remains for research use only and there are few data to support use in an asymptomatic population [16].

The primary objective of this cross-sectional study was to determine the prevalence of ALI in PLHIV residing in VL endemic areas in Bihar. In doing so, we seek to evaluate and correlate results of different diagnostic tools to detect ALI in PLHIV on the ISC. Finally, we determined risk factors for asymptomatic infection in this cohort.

## Methods

### Ethics statement

Informed written consent was obtained from all participants. Ethical approval for this study was granted by Médecins Sans Frontières (MSF) (Ref: 1763). Rajendra Memorial Research Institute of Medical Sciences (Ref: 02/RMRI/EC/2017) and the Liverpool School of Tropical Medicine (LSTM) (Ref: 18–087). The study was prospectively registered at the Clinical Trial Registry India: CTRI/2017/03/008120.

### Study design, population, and recruitment

Over a period of 12 months commencing in May 2018, PLHIV residing in VL endemic villages presenting to three anti-retroviral therapy (ART) centres in one of four VL endemic districts (Saran, Siwan, Muzaffarpur, and Gopalganj) in the state of Bihar, India were screened. Enrolment was open to PLHIV aged ≥ 18 years at any stage of illness, on the condition that they resided in a list of pre-specified villages which had reported at least one VL infection in 2017–18 as per the government kala-azar management information system (KA-MIS). PLHIV with a history of previous treatment for or current diagnosis of symptomatic VL or PKDL were excluded, as was any patient presenting in critical condition or with a severe underlying medical condition whose participation in the study may interfere with immediate medical intervention.

### Sample size

At the time of design, there were few reliable estimate data available on the prevalence of ALI in PLHIV, and none from the Indian context. As such, evidence of ALI in non-immunocompromised individuals living in endemic areas was taken as a point estimate–this ranged from 3.16% to 14% [17–20]. Assuming that PLHIV living within endemic areas would be more likely *a priori* to have ALI due to the degree of lessened immunity, we used an upper threshold of 15% as a likely estimate in PLHIV, in keeping with similar studies in East Africa [21]. A total of 784 and 1352 participants were required to allow for a precision of 2.5% at a confidence level of 95% and 99% respectively. As a lower number of participants would preclude further planned studies on monitoring the progression of ALI patients, we targeted the higher number.

### Recruitment

Over the 12-month recruitment period, the study team rotated between the four ART centres. All patients presenting to the ART centre on the recruitment day were consecutively screened, with a daily maximum target of 20 eligible consenting participants to ensure a manageable workload and allow adequate time to transport samples back to the state capital under cold chain. A screening log was maintained to prevent re-enrolment and to ensure patients who had previously declined to enrol were not reapproached. Sociodemographic data were collected from all enrolled patients, followed by a comprehensive clinical examination.

Blood and urine were then collected for serological and molecular testing, while an immediate rK39 RDT (Kala-azar Detect Rapid Test, Inbios International Inc., WA, USA) was performed on all patients. Any patient meeting the clinical case definition of VL (fever, splenomegaly and a positive rK39 RDT) were immediately referred to a specialist VL-HIV treatment centre in Patna for further assessment and excluded from the study.

HIV-related information including duration of diagnosis, World Health Organization (WHO) clinical staging, and presence of opportunistic infections were collected, as was information on current and past medical conditions. Routine clinical parameters were documented, while nutritional status was determined based on the body mass index (BMI).

Blood and urine were used for determining ALI through serological methods (rK39 RDT and ELISA), molecular methods (qPCR), and the urinary *Leishmania* antigen ELISA (detailed below). Blood was also used for CD4 counts, full blood counts, and HIV viral load. Urine was used for those with CD4 counts <200 cells/mm^3^ to test for lipoarabinomannan (LAM) using the Determine TB-LAM point-of-care tuberculosis assay (Abbott Diagnostics, Lake Bluff, IL, USA). All samples were stored at -80°C and run in batches over the course of the study, with all remaining samples retained in the biobank repository for future research.

ALI was defined as a positive rK39 RDT, rK39 ELISA, and/or qPCR in the absence of clinical symptoms and history of VL or PKDL. A positive urinary *Leishmania* antigen ELISA was not considered ALI in the primary analysis as there were no performance data on the *Leishmania* antigen ELISA in an asymptomatic population and few data in a symptomatic population at the time of study design but was included as ALI in a secondary analysis (detailed extensively in S1–S4 Tables).

### rK39 RDT and ELISA

rK39 RDTs (Kala-azar Detect Rapid Test, Inbios International Inc., WA, USA) were performed using finger-prick capillary blood. Venous blood collected in ethylenediaminetetraacetic acid (EDTA)-treated vacutainers was transported to Patna on ice and stored at 4°C until centrifugation at 3,000rpm for 15 minutes to separate plasma. For the rK39 ELISA, plates were coated overnight at 4°C with rK39 antigen and blocked the next day for 2 hours at 25°C in 1% bovine serum albumin (BSA) in phosphate-buffered saline (PBS). Plates were washed 5 times with wash buffer (0.1% TWEEN-20 in PBS). Following washing, 100μl of each plasma sample, and positive and negative controls diluted 1:400 were incubated on the plate for 30 minutes at 25°C. Plates were washed as before, followed by addition of 100μl of HRP-conjugated secondary antibody for 30 minutes at 25°C. Plates were washed as before and 100μl 3,3’,5,5’-tetramethylbenzidine (TMB) substrate added for 5 minutes in the dark. The reaction was stopped with 1N sulfuric acid. The optical density (OD) was read at 450nm. Results were expressed as the percentage positivity of the positive control.

### Quantitative polymerase chain reaction (qPCR)

DNA was extracted from 100μl peripheral blood using DNeasy Blood and Tissue Kits (Qiagen, Germany) as per the manufacturer’s instructions. Following extraction, appropriate volumes of Quantifast mastermix from the Qiagen QuantiFast PCR Kit, nuclease-free water, kDNA forward and reverse primers and probe were prepared [22]. A total of 1.25μl of extracted DNA was added to each well containing mastermix to a total volume of 12.5μl per well. Samples were loaded onto the BioRad Real-Time PCR Detection System: C1000 Touch and CFX-96 and run at: 1. 95°C for 5 minutes for the initial denaturation; 2. 95°C for 15 seconds at following denaturation steps; 3. 60°C for 30 seconds for annealing and elongation; 4. repetition of steps 2–3, followed by a further 34 cycles. A pre-specified Ct value of ≤35 was considered positive. Although not run in duplicate, PCR was run with negative, positive and extraction controls.

### Leishmania antigen ELISA in urine samples

The *Leishmania* antigen ELISA (Clin-Tech., Guilford, UK) was carried out on urine transported to the Rajendra Memorial Research Institute of Medical Science (RMRIMS) in 15ml falcon tubes on ice and stored at -20°C until testing according to manufacturer’s instructions. Briefly, 10μl of urine was diluted 1 in 20 in assay diluent, applied to a pre-coated plate and incubated at 37°C for 30 minutes. Plates were then washed three times before addition of a secondary detection antibody conjugated to horseradish peroxidase. Following incubation at 37°C for 30 minutes plates were washed as before and TMB substrate added for 30 minutes at room temperature. The reaction was then stopped with addition of a weak acid solution, the plates read within 30 minutes at 450nm and 620nm and the OD values recorded. Any samples with an OD value less than or equal to that of the 2UAU/ml calibrator were considered negative. Concentration of urinary antigen (UAU/ml) was calculated using a standard curve of calibrators and four-parameter curve fitting software, and dilution factor corrected for.

### Statistical analysis

Anonymised data were entered into the database from case report forms by double data entry. The primary outcome measure was the prevalence of ALI. Numerical variables such as age, household size, time to ART, and baseline CD4 counts were grouped into categories as seen in other studies of ALI. A household was defined as a “group of persons who commonly live together and would take their meals from a common kitchen unless the exigencies of work prevented any of them from doing so” as per the Indian census definition of a household. At the time of study design, the latest consensus had found the average household size in Bihar to be 5.5 people per house, and as such, household size was split into <5 or ≥5. Socioeconomic status was divided into five categories based on the BG Prasad Scale [23]. Individuals were classified as severely underweight (BMI<16.5 kg/m^2^), underweight (BMI 16.5–18.5 kg/m^2^), normal (BMI 18.5–25 kg/m^2^), and overweight (>25 kg/m^2^). All continuous variables were summarised as mean (standard deviation) and median (inter-quartile range). Categorical data were presented as counts and percentages. The difference in proportion was analysed by the chi-square or Fisher’s exact test. Student’s t and Wilcoxon rank sum (Mann-Whitney) tests were done to assess differences in mean and median parameters, respectively. The Association of all covariates and the outcome were assessed one by one in univariate analyses. Odds ratio calculations and 95% confidence intervals around proportions in a second step were carried out, and covariates with p-value < 0.2 in the univariate model were included in a logistic regression model. A backward stepwise selection method was applied to determine the independent risk factors for asymptomatic *Leishmania* infection. A p-value ≤ 0.05 was considered a statistically significant difference. Agreement between two tests was calculated using Cohen’s Kappa and the level of agreement was interpreted according to Landis and Koch scale [24]. Data analysis was carried out in R Studio (version 1.3.1056) and SPSS (version 23 Armonk, NY: IBM Corp).

## Results

### Prevalence and determinants of asymptomatic Leishmania infection in PLHIV

**Fig 1:**
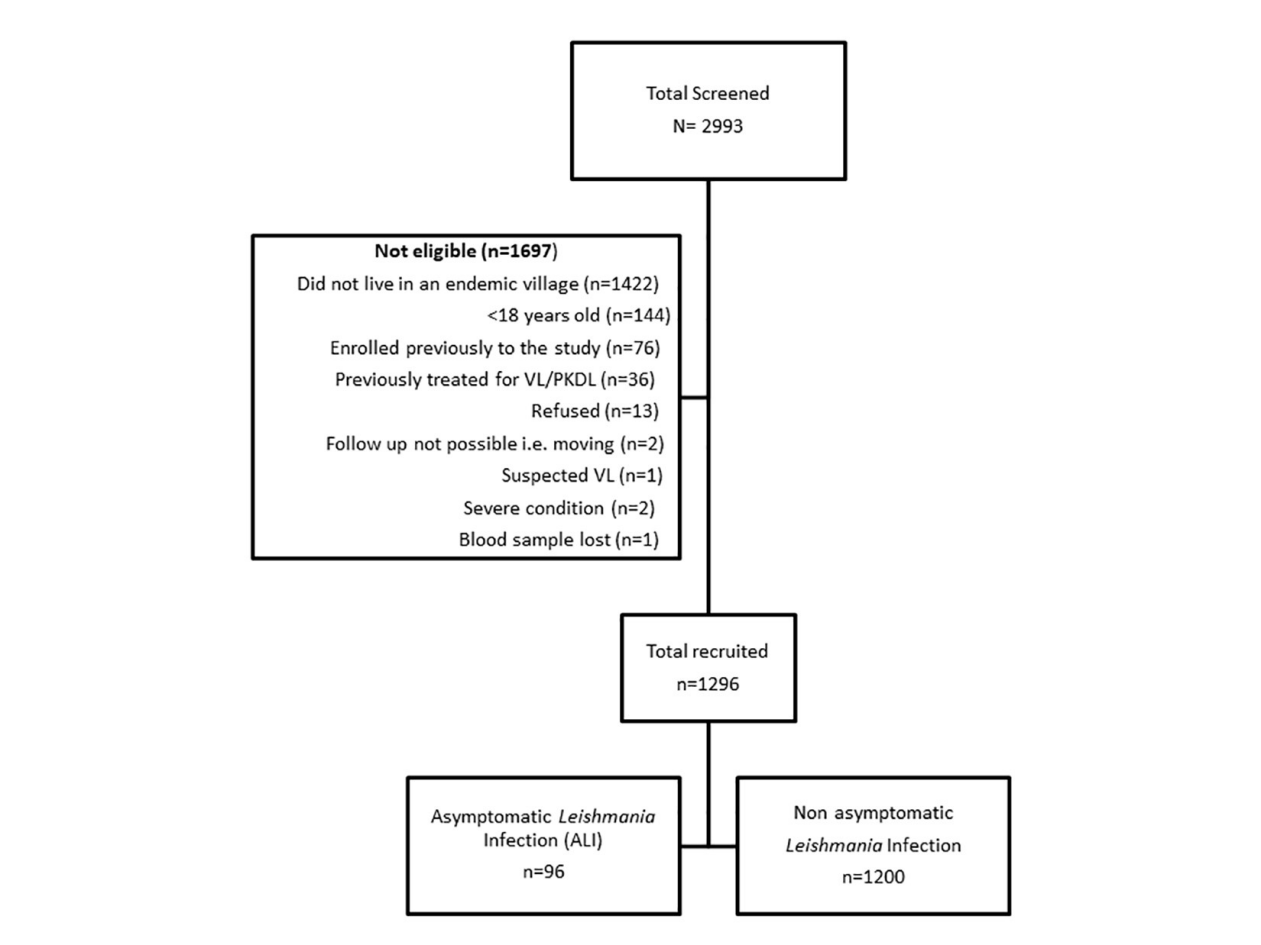
Flow diagram showing recruitment of study participants in Bihar, India between May 2018 and June 2019.

A total of 2,993 individuals were screened, of those, 1,697 individuals did not meet the inclusion criteria and were excluded from the study (Fig 1).

Of the 1,296 PLHIV enrolled in the study, 7.4% (n = 96) met the primary study definition of ALI, detected by rK39 ELISA, rK39 RDT, and/or qPCR. Of the 96 with ALI, the median age was 41 (interquartile range (IQR): 33–50), and 46 (47.9%) were female. Of the ALI and non-ALI (PLHIV negative to all three tests) cohort, 95.8% and 97.8% of patients were on ART respectively, with a median of 32 months on treatment (IQR 12–63). On enrolment, the median CD4 count was 443 cells/mm^3^ (IQR: 303–595) with counts significantly lower in the ALI cohort. Baseline temperature was significantly lower in the non-ALI cohort, although the mean body temperature was within a normal range in both the ALI and non-ALI groups. No significant difference in all other baseline vitals and haematology was seen between the non-asymptomatic and asymptomatic cohort (Table 1).

**Table 1:** Comparison of baseline clinical parameters and haematology results in 1,296 people living with HIV (PLHIV) with and without asymptomatic *Leishmania* infection (ALI) in Bihar, India.

	All Mean (SD) (n = 1296)	ALI (SD) (n = 96)	Non-ALI (SD) (n = 1200)	Mean difference (95% CI)	Sig. (2-tailed)
CD4 (cells/mm^3^)	466 (230)	400 (227)	471 (229)	-71 (-119, -24)	**.003**
Total white cell (count x10^3^/μL)	7.3 (2.4)	7.2 (2.7)	7.3 (2.4)	-0.1 (-0.6, 0.4)	.689
Total red cell (count x10^6^/μL)	3.9 (0.7)	3.9 (0.9)	3.9 (0.7)	0 (-0.1, 0.2)	.938
Haemoglobin (g/dL)	12.2 (2.1)	12 (2.1)	12.2 (2.1)	-0.2 (-0.6, 0.2)	.396
Hematocrit (%)	36.2 (5.6)	35.9 (5.5)	36.2 (5.6)	-0.3 (-1.5, 0.8)	.583
Platelet (x10^3^/μL)	209 (89.1)	213 (101.7)	209 (88.1)	4.2 (-14.3, 22.8)	.654
Lymphocyte (%)	28.1 (8.8)	27.4 (9.7)	28.1 (8.7)	-0.7 (-2.5, 1.2)	.465
Neutrophil (%)	56.5 (12.2)	55.6 (12.9)	56.5 (12.1)	-0.9 (-3.5, 1.6)	.480
Axillary body temperature (°F)	97.5 (1.1)	97.8 (1.0)	97.5 (1.1)	0.3 (0.1, 0.5)	**.007**
Pulse /minute	90.8 (12.2)	92.1 (14.2)	90.7 (12)	1.4 (-1.1, 3.9)	.274
Systolic blood pressure(mmHg)	113.3 (16.1)	113.6 (18.1)	113.3 (15.9)	0.3 (-3, 3.7)	.849
Diastolic blood pressure (mmHg)	72.2 (10.4)	71.9 (9.7)	72.2 (10.5)	-0.4 (-2.5, 1.8)	.738
Oxygen saturation (SpO2) (%)	98.3 (1.5)	98.5 (1.5)	98.3 (1.5)	0.2 (-0.2, 0.5)	.337

Median HIV viral load at baseline in individuals with ALI was found to be 20.0 copies/ml (IQR: 1.0–218.2) (Table 2). HIV viral load was not run in the non-ALI cohort. Of the 139 PLHIV with CD4 count < 200 cells/mm^3^, 18 (13%) were positive by TB-LAM.

**Table 2:** Baseline viral load in 1,296 people living with HIV (PLHIV) with asymptomatic *Leishmania* infection (ALI) in Bihar, India.

Viral Load (copies/ml)	N (%)
Undetectable	39 (44.3)
<150	26 (29.5)
150 to 999	6 (6.8)
1,000 to 9,999	1 (1.1)
10,000 to 99,999	9 (10.2)
100,000 to 1,000,000	6 (6.8)
≥1,000,000	1 (1.1)
Missing	8 (8.3)
Median (IQR)	20 (Undetectable—218)

In a univariate analysis, sex, age, socioeconomic status, type of house, proximity to a pond or livestock, time since last indoor residual spraying (IRS), number of IRS rounds in the last 18 months, contact with a presumptive VL case, contact with a presumptive PKDL case, contact with a cured VL/PKDL case, and the use of bed nets were not significant determinants for ALI (Table 3). Having a household size ≥5 was found to be a risk factor for ALI compared to a smaller household OR = 1.9 (95% CI: 1.1–3.1).

**Table 3:** Household-related risk factors for asymptomatic *Leishmania* infection (ALI) among 1,296 people living with HIV (PLHIV) in Bihar, India.

	All N (%)	Non- ALI N (%)	ALI N (%)	Odds Ratio (95% CI)	P value
Sex					
Female	694 (53.5)	648 (54)	46 (47.9)	Ref	
Male	602 (46.5)	552 (46)	50 (52.1)	1.3 (0.8, 1.9)	0.250
Age (in years)					
18–29	174 (13.4)	159 (13.3)	15 (15.6)	Ref	
30–44	731 (56.4)	686 (57.2)	45 (46.9)	0.7 (0.4, 1.3)	0.240
45–59	329 (25.4)	296 (24.7)	33 (34.4)	1.2 (0.6, 2.2)	0.610
≥ 60	62 (4.8)	59 (4.9)	3 (3.1)	0.5 (0.1, 2)	0.420
Median (IQR)	39 (33–46)	39 (33–46)	41 (33–50)		0.150
Household size					
< 5	395 (30.5)	376 (31.3)	19 (19.8)	Ref	
≥ 5	901 (69.5)	824 (68.7)	77 (80.2)	1.9 (1.1, 3.1)	**0.020**
Median (IQR)	6 (4–7)	6 (4–7)	6 (5–7)		0.271
Socioeconomic status classification					
1 or 2	130 (10.1)	122 (10.2)	8 (8.3)	Ref	
3	333 (25.7)	297 (24.8)	36 (37.5)	1.9 (0.8, 4.1)	0.125
4	513 (39.6)	480 (40.1)	33 (34.4)	1.1 (0.5, 2.3)	0.908
5	318 (24.6)	299 (25.0)	19 (19.8)	1.0 (0.4, 2.3)	0.942
Type of house					
Brick	619 (47.8)	571 (47.6)	48 (50.0)	Ref	
Thatched	281 (21.7)	261 (21.8)	20 (20.8)	0.9 (0.5, 1.6)	0.738
Mud	396 (30.6)	368 (30.7)	28 (29.2)	0.9 (0.6, 1.5)	0.686
Proximity to pond					
No	1,028 (79.3)	945 (78.8)	83 (86.5)	Ref	
Yes	268 (20.7)	255 (21.3)	13 (13.5)	0.6 (0.3, 1.1)	0.066
Proximity to livestock					
No	534 (41.2)	494 (41.2)	40 (41.7)	Ref	
Yes	762 (58.8)	706 (58.8)	56 (58.3)	1.0 (0.6, 1.5)	0.924
Time of last IRS (months)					
< 6	998 (77.0)	915 (76.3)	83 (86.5)	Ref	
Never	144 (11.1)	137 (11.4)	7 (7.3)	0.6 (0.3, 1.2)	0.150
6–12	124 (9.6)	118 (9.8)	6 (6.3)	0.6 (0.2, 1.3)	0.177
> 12	30 (2.3)	30 (2.5)	0 (0)	0 (0, 1.5)	0.154
Number of IRS in last 18 months					
0	140 (10.8)	133 (11.1)	7 (7.3)	Ref	
1	132 (10.2)	128 (10.7)	4 (4.2)	0.6 (0.1, 2.1)	0.540
2	642 (49.5)	593 (49.4)	49 (51.0)	1.6 (0.7, 3.8)	0.270
> 2	382 (29.5)	346 (28.8)	36 (37.5)	2.0 (0.9, 4.6)	0.100
Contact with people with presumptive VL 50 metres around the house					
No/Don’t know	1,236 (95.4)	1,145 (95.4)	91 (94.8)	Ref	
Yes	60 (4.6)	55 (4.6)	5 (5.2)	1.0 (0.4, 2.8)	0.740
Contact with people with presumptive PKDL 50 metres around the house					
No/ Don’t know	1,278 (98.6)	1,184 (98.7)	94 (97.9)	Ref	
Yes	18 (1.4)	16 (1.3)	2 (2.1)	1.6 (0.2, 6.1)	0.780
Contact with people with cured VL/ PKDL 50 metres around the house					
No/ Don’t know	1,262 (97.4)	1,170 (97.5)	92 (95.8)	Ref	
Yes	34 (2.6)	30 (2.5)	4 (4.2)	1.7 (0.5, 4.6)	0.480
Use bed nets while sleeping					
Mostly (>80%)	1,177 (90.8)	1,092 (91.0)	85 (88.5)	Ref	
Never (0%)	30 (2.3)	27 (2.3)	3 (3.1)	1.4 (0.3, 4.8)	0.480
Rarely (1–49%)	22 (1.7)	19 (1.6)	3 (3.1)	2.0 (0.4, 7.1)	0.220
Sometimes (50–80%)	67 (5.2)	62 (5.2)	5 (5.2)	1.0 (0.4, 2.5)	0.941

ART status, WHO stage, concomitant TB infection, ATT status, time since HIV diagnosis, and BMI were not found to be determinants of ALI (Table 4). A CD4 count <100 (OR = 3.1 (95% CI: 1.2–7.6) and a CD4 count between 100–199 (OR = 2.1; 95% CI: 1.1–4.0) were found to be significant independent risk factors for ALI compared to a CD4 count ≥300 (Table 4).

**Table 4:** HIV-related risk factors for asymptomatic *Leishmania* infection (ALI) in 1,296 people living with HIV (PLHIV) in Bihar, India.

	All N (%)	Non- ALI N (%)	ALI N (%)	Odds Ratio (95%CI)	P value
Time on ART					
≥12 months	974 (75.2)	904 (75.3)	70 (72.9)	Ref	
6-<12 months	139 (10.7)	127 (10.6)	12 (12.5)	1.2 (0.6, 2.3)	0.53
<6 months	151 (11.7)	141 (11.8)	10 (10.4)	0.9 (0.4, 1.8)	0.83
Pre-ART	32 (2.5)	28 (2.3)	4 (4.2)	1.8 (0.6, 2.3)	0.41
median (IQR) (excluding Pre-Art)	33 (14–60)	33 (14–60)	32 (12–63)		0.56
WHO clinical Stage					
I	1206 (93.1)	1113 (92.8)	93 (96.9)	Ref	
II	69 (5.3)	68 (5.7)	1 (1)	0.2 (0.004, 1.04)	0.06
III	19 (1.5)	17 (1.4)	2 (2.1)	1.4 (0.2, 5.4)	0.88
IV	2 (0.2)	2 (0.2)	0 (0)	0 (0, 64.8)	1
Tuberculosis treatment status					
Not on anti-tubercular treatment	1144 (88.3)	1057 (88.1)	87 (90.6)	Ref	
History of anti-tubercular treatment	134 (10.3)	128 (10.7)	6 (6.3)	0.6 (0.2, 1.3)	0.190
Currently on anti-tubercular treatment	18 (1.4)	15 (1.3)	3 (3.1)	2.4 (0.4, 8.8)	0.160
Time since HIV diagnosis (years)					
≥ 1	1059 (81.7)	984 (82.0)	75 (78.1)	Ref	
< 1	237 (18.3)	216 (18.0)	21 (21.9)	1.3 (0.8, 2.1)	0.345
BMI (Kg/m^2^)					
<16.5	123 (9.5)	110 (9.2)	13 (13.5)	1.5 (0.8, 2.8)	0.241
16.5-<18.5	292 (22.5)	272 (22.7)	20 (20.8)	0.9 (0.5, 1.5)	0.721
18.5-<25	761 (58.7)	704 (58.7)	57 (59.4)	Ref	
≥25	120 (9.3)	114 (9.5)	6 (6.3)	0.7 (0.3, 1.5)	0.325
Median (IQR)	19.8 (18–22.2)	19.8 (18–22.2)	20.3 (17.3–21.5)		0.538
CD4 (cells / μL)					
≥ 300	978 (75.5)	916 (76.3)	62 (64.6)	Ref	
< 100	35 (12.7)	29 (2.4)	6 (6.3)	3.1 (1.2, 7.6)	**0.012**
100–199	104 (8.0)	91 (7.6)	13 (13.5)	2.1 (1.1, 4.0)	**0.019**
200–299	179 (13.8)	164 (13.7)	15 (15.6)	1.4 (0.8, 2.4)	0.316
Median (IQR)	443 (303–595)	446 (309–598)	367 (223–544)		**0.002**

In a multivariate analysis, a lower CD4 count, and household size ≥ 5 family members were associate with significantly higher risk of ALI. Living in proximity of a pond was the only protective factor for ALI. The final variables in which significance was retained in a multivariable model are shown in Table 5.

**Table 5:** Multivariable risk factor analysis for asymptomatic *Leishmania* infection (ALI) in 1,296 people living with HIV (PLHIV) in Bihar, India.

Variable	aOR (95% CI)	P value
**Household size**		
< 5	**Ref**	
≥ 5	2.3 (1.3, 4.0)	**0.006**
**CD4 group** (cells / μL)		
≥ 300	**Ref**	
<100	3.4 (1.3, 8.8)	**0.012**
100–199	2.4 (1.2, 4.7)	**0.01**
200–299	1.3 (0.7, 2.3)	0.461
**Proximity to a pond**		
No	**Ref**	
Yes	0.5 (0.3, 1.0)	**0.042**

### Effect of urinary antigen on prevalence and determinants of ALI

Prevalence increased to 9.0% (n = 116) when the urinary *Leishmania* antigen ELISA was included in the definition of ALI. Again, having a household size ≥5 was found to be a risk factor for ALI compared to a smaller household OR = 1.8 (95% CI: 1.1–2.8) when urinary *Leishmania* antigen ELISA was included (S1 Table). No other household-related risk factors were identified upon inclusion of the urinary *Leishmania* antigen ELISA (S1 Table).

A CD4 count <100 was no longer significant when urinary *Leishmania* antigen ELISA was included (OR = 2.3 (95% CI: 0.9–5.8, p = 0.06) (S2 Table). A CD4 count between 100–199 (OR = 1.9; 95% CI: 1.1–3.4) remained a significant independent risk factors for ALI compared to a CD4 count ≥300 (Table 4). No other HIV-related risk factors were identified upon inclusion of the urinary *Leishmania* antigen ELISA (S2 Table). The final variables in which significance was retained in a multivariable model are shown in S3 Table. There were few differences in characteristics of individuals testing positive by urinary *Leishmania* antigen ELISA only in comparison to other individuals (S4 Table).

### Diagnostic algorithm to detect asymptomatic Leishmania infection in PLHIV

**Fig 2:**
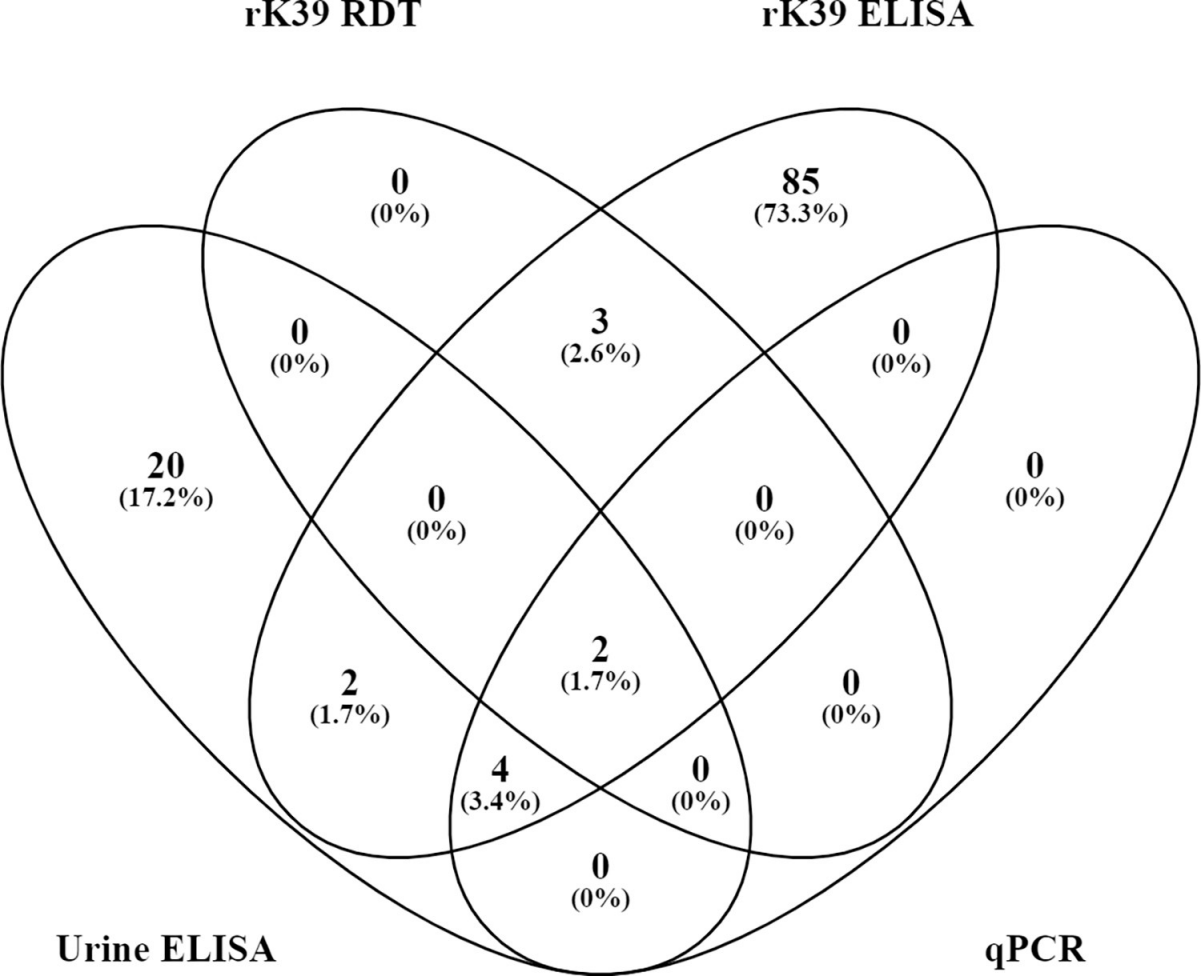
Four assays to detect asymptomatic *Leishmania* infection (ALI) in 1,296 people living with HIV (PLHIV) in Bihar, India.

Ninety-six (7.4%) participants were positive by the rK39 ELISA, 5 (0.4%) by rK39 RDT, and 6 (0.5%) by qPCR, making up the asymptomatic cohort (Table 5). Twenty-eight (2.2%) participants were positive by *Leishmania* antigen ELISA, 20 of which were in addition to the asymptomatic cohort (Table 6). Of the 96 participants, 85 (73.3%) were positive for rK39 ELISA only. Two (1.7%) of the 96 participants tested positive by all four tests. The rK39 ELISA and *Leishmania* antigen ELISA in combination capture all positive participants when the *Leishmania* antigen ELISA is included in the definition of ALI (Fig 2).

**Table 6:** Prevalence of asymptomatic *Leishmania* infection (ALI) in 1,296 people living with HIV (PLHIV) in Bihar, India by serological, molecular, and antigen detection methods.

	N	% (95% CI)
Total recruited	1296	
Total positive by *Leishmania* antigen ELISA	28	2.2 (1.5, 3.1)
Total **ALI** (positive by rK39 RDT or rK39 ELISA or qPCR)	96	7.4 (6.1, 9.0)
Total positive with rK39 RDT	5	0.4 (0.2, 0.9)
Total positive by rK39 ELISA	96	7.4 (6.1, 9.0)
Total positive by qPCR	6	0.5 (0.2, 1.0)

Negligible agreement was seen between the rK39 RDT and rK39 ELISA, rK39 RDT and *Leishmania* antigen ELISA, qPCR and rK39 ELISA, and rK39 ELISA and *Leishmania* antigen ELISA. Weak agreement was seen between rK39 RDT and qPCR, and qPCR and the *Leishmania* antigen ELISA (Table 7).

**Table 7:** Kappa scores and agreement for the rK39 RDT, rK39 ELISA, qPCR, and *Leishmania* antigen ELISA in 96 people living with HIV (PLHIV) with asymptomatic *Leishmania* infection (ALI) in Bihar, India.

Test combination	Agreement (%)	Kappa score	p-value
RK39 RDT and qPCR (n = 9)	99.5	0.361	<0.001
RK39 RDT and rK39 ELISA (n = 96)	93.0	0.092	<0.001
RK39 RDT and *Leishmania* antigen ELISA (n = 31)	90.6	0.115	<0.001
QPCR and rK39 ELISA (n = 96)	93.1	0.110	<0.001
QPCR and *Leishmania* antigen ELISA (n = 28)	98.5	0.348	<0.001
RK39 ELISA and *Leishmania* antigen ELISA (n = 96)	91.7	0.099	<0.001

## Discussion

There has been considerable interest in the role and evolution of ALI over the last decade. No clear consensus exists on this populations role in transmission; one recent xenodiagnostic study from India showed that none of 184 non-HIV infected individuals with ALI were infectious to sandflies; whereas a recent Spanish study demonstrated that sandflies fed on the blood from one ALI-HIV patient, who had been under continuous secondary prophylaxis for leishmaniasis, demonstrated the presence of viable parasites post exposure.[25,26]. Similarly, there remains no clear consensus on the actual definition of asymptomatic infection, and as such caution should be taken when comparing results of different studies reporting prevalence and progression of ALI [27].

Prior to this study the prevalence of ALI in PLHIV residing in VL endemic areas in India was unknown. Prevalence of ALI in this population was 7.4% when detected by a combination of rK39 ELISA, rK39 RDT, and/or qPCR. All individuals with ALI were positive by the rK39 ELISA. A smaller proportion were positive by qPCR (0.5%), and the rK39 RDT (0.4%). All individuals positive by rk39 RDT were also positive by rk39 ELISA. As expected, the rK39 RDT detected a lower number of positive participants compared to the rK39 ELISA, in keeping with the reduced sensitivity of RDTs compared to their equivalent ELISA, and reduced performance in PLHIV as seen in studies in East Africa [14]. Prevalence of ALI increased to 9.0% with the addition of *Leishmania* antigen ELISA, with 20 additional participants identified with the expanded definition of ALI. A low CD4 count and a household size of five individuals or more were found to be risk factors for ALI. Similar to our study findings, a larger household size was identified as a risk factor (OR = 4.4) for *Leishmania* infection in a study by Schenkel *et al*. in Nepal [28]. Household size may be associated with several other factors that may be linked with increased risk of ALI, such as low socioeconomic status. It may be that individuals with low CD4 counts are more susceptible to ALI, or conversely, ALI could lead to lower CD4 counts. A CD4 count <100 was no longer significant when urinary *Leishmania* antigen ELISA was included in the definition of ALI, likely due to a change in proportions of individuals falling within each CD4 count category. As per 2017 estimates of HIV infection in India, 41.2% of *PLHIV* in Bihar were female compared to 53.5% in this study [29].

To the best of our knowledge there has been one other study of ALI in PLHIV in an *L*. *donovani* endemic area [21]. The study in Ethiopia used the lower sensitivity KAtex to detect antigenuria and the DAT to detect anti-*Leishmania* antibodies in addition to PCR and rK39 RDT [21], compared to the *Leishmania* antigen ELISA and the rK39 ELISA used in this study. In Ethiopia, prevalence was found to be 12.8% in males, with being male and a concurrent malaria infection found to be risk factors for ALI [21]. Furthermore, the population had relatively high median CD4 counts (377 cells/mm^3^ (IQR: 250–518)) with generally good overall ART adherence [21]. In this study, median CD4 counts were 443 cells/mm^3^ (IQR: 303–595) with the majority of participants (75.2%) on ART for 12 months or more.

Much of the data on ALI in PLHIV has been collected in areas where *Leishmania infantum* is endemic [20,30,31]. A study in Brazil used PCR, rK39 ELISA, indirect fluorescent antibody test, and an ELISA based on a crude *L*. *infantum* preparation and found the prevalence of ALI in PLHIV to be 20.2% [30]. More recently, a study in Brazil used the rK39 ELISA, rK39 RDT, DAT, KAtex, and PCR to estimate prevalence of ALI in PLHIV, and found prevalence to be 9.1% [31]. Further, a meta-analysis of studies in PLHIV in *L*. *infantum* endemic areas found the prevalence of ALI to be 11.8% [20]. Together these studies provide vital data to inform programmatic policy in a population at risk of poor disease outcomes.

Two participants tested positive by all four tests in combination. As is seen in previous studies of ALI [16,30], weak to negligible agreement was seen between tests. Negligible agreement was seen between the rK39 RDT and *Leishmania* antigen ELISA, qPCR and rK39 ELISA, and rK39 ELISA and *Leishmania* antigen ELISA, which may be expected given the tests in these combinations detect anti-*Leishmania* antibodies in comparison to assays which detect active infection [32]. Better, albeit weak agreement was seen between the rK39 RDT and qPCR possibly due a lower sensitivity of the rK39 RDT and a higher specificity of qPCR. There was similarly better, but weak agreement between qPCR and the *Leishmania* antigen ELISA, in keeping with them both detecting active infection. To the best of our knowledge, this is the first study to use the *Leishmania* antigen ELISA to detect ALI in PLHIV. We acknowledge that the specificity of the tests falls below 100% and as such there will be false positives within the data.

This study is limited by the absence of follow-up data, although follow-up data is due to be presented in the next year. This study was further limited by the lack of data on the *Leishmania* antigen ELISA in an asymptomatic population at the time of study conception, and as such was not included in the primary definition of ALI. Further tests could have included the DAT given its wide use in other studies of ALI.

Here, we provide estimates of prevalence and determinants of ALI in PLHIV in a VL endemic region of India. Work is ongoing to determine rate and risk factors for progression to VL in this population. Further longitudinal data are required to estimate incidence of ALI in this population.

## Supporting information

S1 STROBE Checklist STROBE Statement—checklist of items that should be included in reports of observational studies. (DOCX)

S1 Table Household related risk factors for asymptomatic *Leishmania* infection (ALI) in PLHIV including the *Leishmania* antigen ELISA in addition to qPCR, rK39 ELISA and RDT in the definition of ALI. (DOCX)

S2 Table HIV related risk factors for asymptomatic *Leishmania* infection (ALI) in PLHIV including the *Leishmania* antigen ELISA in addition to qPCR, rK39 ELISA and RDT in the definition of ALI. (DOCX)

S3 Table Multivariable risk factor analysis for ALI in PLHIV including the *Leishmania* antigen ELISA in addition to qPCR, rK39 ELISA and RDT in the definition of ALI. (DOCX)

S4 Table Differences in baseline characteristics of individuals testing positive by urinary *Leishmania* antigen ELISA only in comparison other individuals. (DOCX)
