# Supplementary material for: Prevalence and determinants of asymptomatic Leishmania infection in HIV-infected individuals living within visceral leishmaniasis endemic areas of Bihar, India
Source: PLoS Negl Trop Dis. 2022 Aug 30;16(8):e0010718. doi: 10.1371/journal.pntd.0010718 (PMC9467307; doi:10.1371/journal.pntd.0010718)
Supplement: S1 STROBE Checklist — (DOCX) [file pntd.0010718.s001.docx]

STROBE Statement—checklist of items that should be included in reports of observational studies

|  | Item No. | Recommendation | Page  No. | Relevant text from manuscript |
| --- | --- | --- | --- | --- |
| **Title and abstract** | 1 | (*a*) Indicate the study’s design with a commonly used term in the title or the abstract | 2 | We conducted a cross-sectional survey of PLHIV ≥18 years of age with no history or current diagnosis of VL or post kala-azar dermal leishmaniasis (PKDL) at anti-retroviral therapy centres within VL endemic districts of Bihar. |
|  |  | (*b*) Provide in the abstract an informative and balanced summary of what was done and what was found | 2 | We conducted a cross-sectional survey of PLHIV ≥18 years of age with no history or current diagnosis of VL or post kala-azar dermal leishmaniasis (PKDL) at anti-retroviral therapy centres within VL endemic districts of Bihar. ALI was defined as a positive rK39 enzyme-linked immunosorbent assay (ELISA), rK39 rapid diagnostic test (RDT) and/or quantitative polymerase chain reaction (qPCR). Additionally, the urinary *Leishmania* antigen ELISA was evaluated. Determinants for ALI were established using logistic regression and agreement between diagnostic tests calculated using Cohen’s Kappa. A total of 1,296 PLHIV enrolled in HIV care, 694 (53.6%) of whom were female and a median age of 39 years (interquartile range 33–46), were included in the analysis. Baseline prevalence of ALI was 7.4% (n=96). All 96 individuals were positive by rK39 ELISA, while 0.5% (n=6) and 0.4% (n=5) were positive by qPCR and rK39 RDT, respectively. Independent risk factors for ALI were CD4 counts <100 (OR 3.1; 95% CI 1.2–7.6) and CD4 counts 100-199 (OR=2.1; 95% CI: 1.1-4.0) compared to CD4 counts ≥300, and a household size ≥5 (OR=1.9; 95% CI: 1.1-3.1). A total of 2.2% (n=28) participants were positive by Leishmania antigen ELISA, detecting 20 additional participants to the asymptomatic cohort. |
| Introduction | | | |  |
| Background/rationale | 2 | Explain the scientific background and rationale for the investigation being reported | 4 and 5 | Furthermore, where asymptomatic Leishmania infection (ALI) may represent an anthroponotic reservoir on the Indian subcontinent (10), data on prevalence and determinants for ALI and guidelines on optimal screening algorithms in PLHIV are absent. This evidence gap is potentially important; the majority of patients diagnosed with VL-HIV present at a late stage with advanced HIV; assuming a reasonably high progression from asymptomatic to symptomatic VL infection in PLHIV, the utility of a tool that could potentially be used to identify the subclinical form earlier could be of major benefit in the early detection and management of this co-infection. Such screen-and-treat strategies in East Africa have been conceptionally described elsewhere (11).  As the effort to eliminate VL as a public health problem has progressed substantially in the ISC, the proportion of patients with VL-HIV has increased both in absolute numbers and as a proportion of all VL cases. Indeed, more recent analysis of the epidemiological spread and impact of VL-HIV has suggested that the presence of VL-HIV cases was associated with a greater than two-fold increase in VL incidence at the village level, with an incidence risk ratio similar to that of post kala-azar dermal leishmaniasis (PKDL) (12). As such, establishing the scale of asymptomatic infections in HIV patients may contribute significantly to improved programmatic policy in sustaining elimination targets. |
| Objectives | 3 | State specific objectives, including any prespecified hypotheses | 5 | The primary objective of this cross-sectional study was to determine the prevalence of ALI in PLHIV residing in VL endemic areas in Bihar. In doing so, we seek to evaluate and correlate results of different diagnostic tools to detect ALI in PLHIV on the ISC. Finally, we determined risk factors for asymptomatic infection in this cohort. |
| Methods | | | |  |
| Study design | 4 | Present key elements of study design early in the paper | 6 | Over a period of 12 months commencing in May 2018, PLHIV residing in VL endemic villages who presented to anti-retroviral therapy (ART) centres in one of four VL endemic districts (Saran, Siwan, Muzaffarpur, and Gopalganj) in the state of Bihar, India were enrolled in the study. Enrolment was open to PLHIV aged ≥ 18 years at any stage of illness, on condition that they resided in a list of pre-specified villages which had reported at least one VL infection in 2017-18 as per the government kala-azar management information system (KA-MIS). PLHIV with a history of previous treatment for, or current diagnosis of symptomatic VL or PKDL were excluded, as was any patient presenting in critical condition or with a severe underlying medical condition whose participation in the study may interfere with immediate medical intervention. |
| Setting | 5 | Describe the setting, locations, and relevant dates, including periods of recruitment, exposure, follow-up, and data collection | 6 and 7 | Over a period of 12 months commencing in May 2018, PLHIV residing in VL endemic villages who presented to anti-retroviral therapy (ART) centres in one of four VL endemic districts (Saran, Siwan, Muzaffarpur, and Gopalganj) in the state of Bihar, India were enrolled in the study.  Over the 12-month recruitment period, the study team rotated between the four ART centres. All patients presenting to the ART centre on the recruitment day were consecutively screened, with a daily maximum target of 20 eligible consenting participants to ensure manageable workload and allow adequate time to transport samples back to the state capital under cold chain. A screening log was maintained to prevent re-enrolment, and to ensure patients who had previously declined to enrol were not reapproached. Sociodemographic data were collected from all enrolled patients, followed by a comprehensive clinical examination. |
| Participants | 6 | (*a*) *Cohort study*—Give the eligibility criteria, and the sources and methods of selection of participants. Describe methods of follow-up  *Case-control study*—Give the eligibility criteria, and the sources and methods of case ascertainment and control selection. Give the rationale for the choice of cases and controls  *Cross-sectional study*—Give the eligibility criteria, and the sources and methods of selection of participants | 6 and 7 | Over a period of 12 months commencing in May 2018, PLHIV residing in VL endemic villages who presented to anti-retroviral therapy (ART) centres in one of four VL endemic districts (Saran, Siwan, Muzaffarpur, and Gopalganj) in the state of Bihar, India were enrolled in the study. Enrolment was open to PLHIV aged ≥ 18 years at any stage of illness, on condition that they resided in a list of pre-specified villages which had reported at least one VL infection in 2017-18 as per the government kala-azar management information system (KA-MIS). PLHIV with a history of previous treatment for, or current diagnosis of symptomatic VL or PKDL were excluded, as was any patient presenting in critical condition or with a severe underlying medical condition whose participation in the study may interfere with immediate medical intervention. Over the 12-month recruitment period, the study team rotated between the four ART centres. All patients presenting to the ART centre on the recruitment day were consecutively screened, with a daily maximum target of 20 eligible consenting participants to ensure manageable workload and allow adequate time to transport samples back to the state capital under cold chain. A screening log was maintained to prevent re-enrolment, and to ensure patients who had previously declined to enrol were not reapproached. Sociodemographic data were collected from all enrolled patients, followed by a comprehensive clinical examination. |
|  |  | (*b*) *Cohort study*—For matched studies, give matching criteria and number of exposed and unexposed  *Case-control study*—For matched studies, give matching criteria and the number of controls per case |  |  |
| Variables | 7 | Clearly define all outcomes, exposures, predictors, potential confounders, and effect modifiers. Give diagnostic criteria, if applicable | 7 | Blood and urine were then collected for serological and molecular testing, while an immediate rK39 RDT (Kala-azar Detect Rapid Test, Inbios International Inc., WA, USA) was performed on all patients. Any patient meeting the clinical case definition of VL (fever, splenomegaly and a positive rK39 RDT) were immediately referred to a specialist VL-HIV treatment centre in Patna for further assessment and excluded from the study if confirmed to be symptomatic VL.  Socioeconomic status was divided into five categories based on the BG Prasad Scale (21). HIV-related information including duration of diagnosis, World Health Organization (WHO) clinical staging, and presence of opportunistic infections was collected, as was information on current and past medical conditions. Routine clinical parameters were documented, while nutritional status was determined based on the body mass index (BMI). Individuals were classified as severely underweight (BMI<16.5 kg/m^2^), underweight (BMI 16.5-18.5 kg/m^2^), normal (BMI 18.5-25 kg/m2), and overweight (>25 kg/m2).  Blood and urine were used for determining ALI through serological methods (rK39 RDT and ELISA), molecular methods (qPCR), and the urinary *Leishmania* antigen ELISA (detailed below). Blood was also used for CD4 counts, full blood counts, and HIV viral load. Urine was used for those with CD4 counts <200 cells/mm^3^to test for lipoarabinomannan (LAM) using the Determine TB-LAM point-of-care tuberculosis assay (Abbott Diagnostics, Lake Bluff, IL, USA). |
| Data sources/ measurement | 8* | For each variable of interest, give sources of data and details of methods of assessment (measurement). Describe comparability of assessment methods if there is more than one group | 7 and 8 | Sociodemographic data were collected from all enrolled patients, followed by a comprehensive clinical examination.  Blood and urine were then collected for serological and molecular testing, while an immediate rK39 RDT (Kala-azar Detect Rapid Test, Inbios International Inc., WA, USA) was performed on all patients. Any patient meeting the clinical case definition of VL (fever, splenomegaly and a positive rK39 RDT) were immediately referred to a specialist VL-HIV treatment centre in Patna for further assessment and excluded from the study if confirmed to be symptomatic VL.  Socioeconomic status was divided into five categories based on the BG Prasad Scale (21). HIV-related information including duration of diagnosis, World Health Organization (WHO) clinical staging, and presence of opportunistic infections was collected, as was information on current and past medical conditions. Routine clinical parameters were documented, while nutritional status was determined based on the body mass index (BMI). Individuals were classified as severely underweight (BMI<16.5 kg/m^2^), underweight (BMI 16.5-18.5 kg/m^2^), normal (BMI 18.5-25 kg/m2), and overweight (>25 kg/m2).  Blood and urine were used for determining ALI through serological methods (rK39 RDT and ELISA), molecular methods (qPCR), and the urinary *Leishmania* antigen ELISA (detailed below). Blood was also used for CD4 counts, full blood counts, and HIV viral load. Urine was used for those with CD4 counts <200 cells/mm^3^to test for lipoarabinomannan (LAM) using the Determine TB-LAM point-of-care tuberculosis assay (Abbott Diagnostics, Lake Bluff, IL, USA). All samples were stored at -80°C and run in batches over the course of the study, with all remaining samples retained in the biobank repository for future research.  ALI was defined as a positive rK39 RDT, rK39 ELISA, and/or qPCR in the absence of clinical symptoms and history of VL or PKDL. A positive urinary *Leishmania* antigen ELISA was not considered ALI in the primary analysis as there were no performance data on the *Leishmania* antigen ELISA in an asymptomatic population and few data in a symptomatic population at the time of study design but was included as ALI in a secondary analysis (detailed extensively in supplementary materials). |
| Bias | 9 | Describe any efforts to address potential sources of bias | 7 and 10 | Over the 12-month recruitment period, the study team rotated between the four ART centres. All patients presenting to the ART centre on the recruitment day were consecutively screened, with a daily maximum target of 20 eligible consenting participants to ensure manageable workload and allow adequate time to transport samples back to the state capital under cold chain. A screening log was maintained to prevent re-enrolment, and to ensure patients who had previously declined to enrol were not reapproached. Sociodemographic data were collected from all enrolled patients, followed by a comprehensive clinical examination.  Data analysis was carried out in R Studio (version 1.3.1056) and SPSS (version 23). Continuous variables were summarised as mean (standard deviation) and median (inter-quartile range). Categorical data was presented as counts and percentages. Difference in proportion was analysed by the chi-square or Fisher’s exact test. Association of all covariates and the outcome was assessed one by one in bivariate analyses. Odds ratio calculations were carried out along with 95% confidence intervals around proportions in a second step, covariates with p-value < 0.2 in the bivariate model were included in a logistic regression model. A backward stepwise selection method was applied to determine the independent risk factors for asymptomatic *Leishmania* infection. A p-value ≤ 0.05 was considered a statistically significant difference. The values of Cohen’s k coefficients were interpreted according to Landis and Koch (22). |
| Study size | 10 | Explain how the study size was arrived at | 6 | At the time of design, there were no reliable estimate data available on the prevalence of ALI in PLHIV, and none from the Indian context. As such, evidence of ALI in non-immunocompromised individuals living in endemic areas was taken as a point estimate – this ranged from 3.16% to 14% (16–19). Assuming that PLHIV living within endemic areas would be more likely *a priori* to have ALI due to the degree of lessened immunity, we used an upper threshold of 15% as a likely estimate in PLHIV, in keeping with similar studies in East Africa (20). A total of 784 and 1352 participants were required to allow for a precision of 2.5% at a confidence level of 95% and 99% respectively. As a lower number of participants would preclude further planned studies on monitoring progression of ALI patients, we targeted the higher number. |

Continued on next page

| Quantitative variables | 11 | Explain how quantitative variables were handled in the analyses. If applicable, describe which groupings were chosen and why | 7, 8, 9, and 10 | Blood and urine were then collected for serological and molecular testing, while an immediate rK39 RDT (Kala-azar Detect Rapid Test, Inbios International Inc., WA, USA) was performed on all patients. Any patient meeting the clinical case definition of VL (fever, splenomegaly and a positive rK39 RDT) were immediately referred to a specialist VL-HIV treatment centre in Patna for further assessment and excluded from the study if confirmed to be symptomatic VL.  Socioeconomic status was divided into five categories based on the BG Prasad Scale (21). HIV-related information including duration of diagnosis, World Health Organization (WHO) clinical staging, and presence of opportunistic infections was collected, as was information on current and past medical conditions. Routine clinical parameters were documented, while nutritional status was determined based on the body mass index (BMI). Individuals were classified as severely underweight (BMI<16.5 kg/m^2^), underweight (BMI 16.5-18.5 kg/m^2^), normal (BMI 18.5-25 kg/m2), and overweight (>25 kg/m2).  Blood and urine were used for determining ALI through serological methods (rK39 RDT and ELISA), molecular methods (qPCR), and the urinary *Leishmania* antigen ELISA (detailed below). Blood was also used for CD4 counts, full blood counts, and HIV viral load. Urine was used for those with CD4 counts <200 cells/mm^3^to test for lipoarabinomannan (LAM) using the Determine TB-LAM point-of-care tuberculosis assay (Abbott Diagnostics, Lake Bluff, IL, USA). All samples were stored at -80°C and run in batches over the course of the study, with all remaining samples retained in the biobank repository for future research.  ALI was defined as a positive rK39 RDT, rK39 ELISA, and/or qPCR in the absence of clinical symptoms and history of VL or PKDL. A positive urinary *Leishmania* antigen ELISA was not considered ALI in the primary analysis as there were no performance data on the *Leishmania* antigen ELISA in an asymptomatic population and few data in a symptomatic population at the time of study design but was included as ALI in a secondary analysis (detailed extensively in supplementary materials).  Data analysis was carried out in R Studio (version 1.3.1056) and SPSS (version 23). Continuous variables were summarised as mean (standard deviation) and median (inter-quartile range). Categorical data was presented as counts and percentages. Difference in proportion was analysed by the chi-square or Fisher’s exact test. Association of all covariates and the outcome was assessed one by one in bivariate analyses. Odds ratio calculations were carried out along with 95% confidence intervals around proportions in a second step, covariates with p-value < 0.2 in the bivariate model were included in a logistic regression model. A backward stepwise selection method was applied to determine the independent risk factors for asymptomatic *Leishmania* infection. A p-value ≤ 0.05 was considered a statistically significant difference. The values of Cohen’s k coefficients were interpreted according to Landis and Koch (22). |
| --- | --- | --- | --- | --- |
| Statistical methods | 12 | (*a*) Describe all statistical methods, including those used to control for confounding | 9 and 10 | Data analysis was carried out in R Studio (version 1.3.1056) and SPSS (version 23). Continuous variables were summarised as mean (standard deviation) and median (inter-quartile range). Categorical data was presented as counts and percentages. Difference in proportion was analysed by the chi-square or Fisher’s exact test. Association of all covariates and the outcome was assessed one by one in bivariate analyses. Odds ratio calculations were carried out along with 95% confidence intervals around proportions in a second step, covariates with p-value < 0.2 in the bivariate model were included in a logistic regression model. A backward stepwise selection method was applied to determine the independent risk factors for asymptomatic *Leishmania* infection. A p-value ≤ 0.05 was considered a statistically significant difference. The values of Cohen’s k coefficients were interpreted according to Landis and Koch (22). |
|  |  | (*b*) Describe any methods used to examine subgroups and interactions | 9 and 10 | Data analysis was carried out in R Studio (version 1.3.1056) and SPSS (version 23). Continuous variables were summarised as mean (standard deviation) and median (inter-quartile range). Categorical data was presented as counts and percentages. Difference in proportion was analysed by the chi-square or Fisher’s exact test. Association of all covariates and the outcome was assessed one by one in bivariate analyses. Odds ratio calculations were carried out along with 95% confidence intervals around proportions in a second step, covariates with p-value < 0.2 in the bivariate model were included in a logistic regression model. A backward stepwise selection method was applied to determine the independent risk factors for asymptomatic *Leishmania* infection. A p-value ≤ 0.05 was considered a statistically significant difference. The values of Cohen’s k coefficients were interpreted according to Landis and Koch (22). |
|  |  | (*c*) Explain how missing data were addressed | 9 and 10 | Data analysis was carried out in R Studio (version 1.3.1056) and SPSS (version 23). Continuous variables were summarised as mean (standard deviation) and median (inter-quartile range). Categorical data was presented as counts and percentages. Difference in proportion was analysed by the chi-square or Fisher’s exact test. Association of all covariates and the outcome was assessed one by one in bivariate analyses. Odds ratio calculations were carried out along with 95% confidence intervals around proportions in a second step, covariates with p-value < 0.2 in the bivariate model were included in a logistic regression model. A backward stepwise selection method was applied to determine the independent risk factors for asymptomatic *Leishmania* infection. A p-value ≤ 0.05 was considered a statistically significant difference. The values of Cohen’s k coefficients were interpreted according to Landis and Koch (22). |
|  |  | (*d*) *Cohort study*—If applicable, explain how loss to follow-up was addressed  *Case-control study*—If applicable, explain how matching of cases and controls was addressed  *Cross-sectional study*—If applicable, describe analytical methods taking account of sampling strategy | NA | NA |
|  |  | (*e*) Describe any sensitivity analyses | NA | NA |
| Results | | | | |
| Participants | 13* | (a) Report numbers of individuals at each stage of study—eg numbers potentially eligible, examined for eligibility, confirmed eligible, included in the study, completing follow-up, and analysed | 10 | A total of 1,589 individuals were screened, of those, 293 individuals did not meet the inclusion criteria and were excluded from the study. Of the 1,296 PLHIV enrolled in the study, 7.4% (n=96) met the primary study definition of ALI, detected by rK39 ELISA, rK39 RDT, and/or qPCR. |
|  |  | (b) Give reasons for non-participation at each stage | 10 | A total of 1,589 individuals were screened, of those, 293 individuals did not meet the inclusion criteria and were excluded from the study. Of the 1,296 PLHIV enrolled in the study, 7.4% (n=96) met the primary study definition of ALI, detected by rK39 ELISA, rK39 RDT, and/or qPCR. |
|  |  | (c) Consider use of a flow diagram | NA | NA |
| Descriptive data | 14* | (a) Give characteristics of study participants (eg demographic, clinical, social) and information on exposures and potential confounders | 10 | Of the 1,296 PLHIV enrolled in the study, 7.4% (n=96) met the primary study definition of ALI, detected by rK39 ELISA, rK39 RDT, and/or qPCR. Of the 96 with ALI, the median age was 41 (inter quartile range (IQR): 33-50), and 46 (47.9%) were female. Of the ALI and non-ALI (PLHIV negative to all 3 tests) cohort, 95.8% and 97.8% of patients were on ART respectively, with a median of 32 months on treatment (IQR 12-63). On enrolment, the median CD4 count was 443 cells/mm^3^ (IQR: 303-595) with counts significantly lower in the ALI cohort. Baseline temperature was significantly lower in the non-ALI cohort. No significant difference on all other baseline vitals and haematology was seen between the non-asymptomatic and asymptomatic cohort (Table 1). |
|  |  | (b) Indicate number of participants with missing data for each variable of interest | All Tables | N reported in all tables. |
|  |  | (c) *Cohort study*—Summarise follow-up time (eg, average and total amount) | NA | NA |
| Outcome data | 15* | *Cohort study*—Report numbers of outcome events or summary measures over time | NA | NA |
|  |  | *Case-control study—*Report numbers in each exposure category, or summary measures of exposure | NA | NA |
|  |  | *Cross-sectional study—*Report numbers of outcome events or summary measures | 10, Tables 1-7 | Of the 1,296 PLHIV enrolled in the study, 7.4% (n=96) met the primary study definition of ALI, detected by rK39 ELISA, rK39 RDT, and/or qPCR. Table 1-7. |
| Main results | 16 | (*a*) Give unadjusted estimates and, if applicable, confounder-adjusted estimates and their precision (eg, 95% confidence interval). Make clear which confounders were adjusted for and why they were included | 12, 15, 17, and 18, Tables 1-7. | In a univariate analysis, sex, age, socioeconomic status, type of house, proximity to a pond or livestock, time since last indoor residual spraying (IRS), number of IRS rounds in the last 18 months, contact with a presumptive VL case, contact with a presumptive PKDL case, contact with a cured VL/PKDL case, and the use of bed nets were not significant determinants for ALI (Table 3). Living within the district of Gopalganj appeared to be protective of having an ALI OR=0.1 (95% CI: 0.002-0.5) compared to Saran district. Having a household size ≥5 was found to be a risk factor for ALI compared to a smaller household OR=1.9 (95% CI: 1.1-3.1).  ART status, WHO stage, concomitant TB infection, ATT status, time since HIV diagnosis, and BMI were not found to be determinants of ALI (Table 4). A CD4 count <100 (OR=3.1 (95% CI: 1.2-7.6) and a CD4 count between 100-199 (OR=2.1; 95% CI: 1.1-4.0) were found to be significant independent risk factors for ALI compared to a CD4 count ≥300 (Table 4).  Prevalence increased to 9.0% (n=116) when the urinary *Leishmania* antigen ELISA was included in the definition of ALI. With the inclusion of the urinary *Leishmania* antigen ELISA, living within the district of Gopalganj remained protective of having an ALI OR=0.1 (95% CI: 0.02-0.5) compared to Saran district (S1 Table). Again, having a household size ≥5 was found to be a risk factor for ALI compared to a smaller household OR=1.8 (95% CI: 1.1-2.8) when urinary *Leishmania* antigen ELISA was included (S1 Table). No other household-related risk factors were identified upon inclusion of the urinary *Leishmania* antigen ELISA (S1 Table).  A CD4 count <100 was no longer significant when urinary *Leishmania* antigen ELISA was included (OR=2.3 (95% CI: 0.9-5.8, p=0.06) (S2 Table). A CD4 count between 100-199 (OR=1.9; 95% CI: 1.1-3.4) remained a significant independent risk factors for ALI compared to a CD4 count ≥300 (Table 4). No other HIV-related risk factors were identified upon inclusion of the urinary *Leishmania* antigen ELISA (S2 Table).  Tables 1-7. |
|  |  | (*b*) Report category boundaries when continuous variables were categorized | NA | NA |
|  |  | (*c*) If relevant, consider translating estimates of relative risk into absolute risk for a meaningful time period | NA | NA |

Continued on next page

| Other analyses | 17 | Report other analyses done—eg analyses of subgroups and interactions, and sensitivity analyses | 18 and 19 | Prevalence increased to 9.0% (n=116) when the urinary *Leishmania* antigen ELISA was included in the definition of ALI. With the inclusion of the urinary *Leishmania* antigen ELISA, living within the district of Gopalganj remained protective of having an ALI OR=0.1 (95% CI: 0.02-0.5) compared to Saran district (S1 Table). Again, having a household size ≥5 was found to be a risk factor for ALI compared to a smaller household OR=1.8 (95% CI: 1.1-2.8) when urinary *Leishmania* antigen ELISA was included (S1 Table). No other household-related risk factors were identified upon inclusion of the urinary *Leishmania* antigen ELISA (S1 Table).  A CD4 count <100 was no longer significant when urinary *Leishmania* antigen ELISA was included (OR=2.3 (95% CI: 0.9-5.8, p=0.06) (S2 Table). A CD4 count between 100-199 (OR=1.9; 95% CI: 1.1-3.4) remained a significant independent risk factors for ALI compared to a CD4 count ≥300 (Table 4). No other HIV-related risk factors were identified upon inclusion of the urinary *Leishmania* antigen ELISA (S2 Table). Ninety-six (7.4%) participants were positive by the rK39 ELISA, 5 (0.4%) by rK39 RDT, and 6 (0.5%) by qPCR, making up the asymptomatic cohort (Table 5). Twenty-eight (2.2%) participants were positive by *Leishmania* antigen ELISA, 20 of which were in addition to the asymptomatic cohort (Table 5). Of the 96 participants, 85 (73.3%) were positive for rK39 ELISA only. Two (1.7%) of the 96 participants tested positive by all four tests. The rK39 ELISA and *Leishmania* antigen ELISA in combination capture all positive participants when the *Leishmania* antigen ELISA is included in the definition of ALI (Fig 1).  Negligible agreement was seen between the rK39 RDT and rK39 ELISA, rK39 RDT and *Leishmania* antigen ELISA, qPCR and rK39 ELISA, and rK39 ELISA and *Leishmania* antigen ELISA. Weak agreement was seen between rK39 RDT and qPCR, and qPCR and the *Leishmania* antigen ELISA (Table 6). |
| --- | --- | --- | --- | --- |
| Discussion | | | | |
| Key results | 18 | Summarise key results with reference to study objectives | 20 and 21 | Prior to this study the prevalence of asymptomatic *Leishmania* infection in PLHIV residing in VL endemic areas in India was unknown. Prevalence of ALI in this population was 7.4% when detected by a combination of rK39 ELISA, rK39 RDT, and/or qPCR. All individuals with ALI were positive by the rK39 ELISA. A smaller proportion were positive by qPCR (0.5%), and the rK39 RDT (0.4%). As expected, the rK39 RDT detected a lower number of positive participants compared to the rK39 ELISA, in keeping with the reduced sensitivity of RDTs compared to the equivalent ELISA, and reduced performance in PLHIV as seen in studies in East Africa (13). Prevalence of ALI increased to 9.0% with the addition of *Leishmania* antigen ELISA, with 20 additional participants identified with the expanded definition of ALI. A low CD4 count and a household size of five individuals or more were found to be risk factors for ALI. Conversely, living within the district of Gopalganj was found to be protective for ALI. Gopalganj has lower rates of *Leishmania* infection than other districts in the study. As per 2017 estimates of HIV infection in India, 41.2% of *PLHIV* in Bihar were female compared to 53.5% in this study (26). |
| Limitations | 19 | Discuss limitations of the study, taking into account sources of potential bias or imprecision. Discuss both direction and magnitude of any potential bias | 22 | This study is limited by absence of follow-up data, although follow-up data is due to be presented in the next year. This study was further limited by the lack of data on the *Leishmania* antigen ELISA in an asymptomatic population at the time of study conception, and as such was not included in the primary definition of ALI. Further tests could have included the DAT given its wide use in other studies of ALI. |
| Interpretation | 20 | Give a cautious overall interpretation of results considering objectives, limitations, multiplicity of analyses, results from similar studies, and other relevant evidence | 20, 21, and 22 | Prior to this study the prevalence of ALI in PLHIV residing in VL endemic areas in India was unknown. Prevalence of ALI in this population was 7.4% when detected by a combination of rK39 ELISA, rK39 RDT, and/or qPCR. All individuals with ALI were positive by the rK39 ELISA. A smaller proportion were positive by qPCR (0.5%), and the rK39 RDT (0.4%). As expected, the rK39 RDT detected a lower number of positive participants compared to the rK39 ELISA, in keeping with the reduced sensitivity of RDTs compared to their equivalent ELISA, and reduced performance in PLHIV as seen in studies in East Africa (13). Prevalence of ALI increased to 9.0% with the addition of *Leishmania* antigen ELISA, with 20 additional participants identified with the expanded definition of ALI. A low CD4 count and a household size of five individuals or more were found to be risk factors for ALI. Conversely, living within the district of Gopalganj was found to be protective for ALI. Gopalganj has lower rates of *Leishmania* infection than other districts in the study. As per 2017 estimates of HIV infection in India, 41.2% of *PLHIV* in Bihar were female compared to 53.5% in this study (26).  Two participants tested positive by all four tests in combination. As is seen in previous studies of ALI (15,27), weak to negligible agreement was seen between tests. Negligible agreement was seen between the rK39 RDT and *Leishmania* antigen ELISA, qPCR and rK39 ELISA, and rK39 ELISA and *Leishmania* antigen ELISA, which may be expected given the tests in these combinations detect anti-*Leishmania* antibodies in comparison to assays which detect active infection (29). Better, albeit weak agreement was seen between the rK39 RDT and qPCR possibly due a lower sensitivity of the rK39 RDT and a higher specificity of qPCR. There was similarly better, but weak agreement between qPCR and the *Leishmania* antigen ELISA, in keeping with them both detecting active infection. To the best of our knowledge, this is the first study to use the *Leishmania* antigen ELISA to detect ALI in PLHIV. |
| Generalisability | 21 | Discuss the generalisability (external validity) of the study results | 20, 21, and 22 | To the best of our knowledge there has been one other study of ALI in PLHIV in an *L. donovani* endemic area (20). The study in Ethiopia used the lower sensitivity KAtex to detect antigenuria and the DAT to detect anti-*Leishmania* antibodies in addition to PCR and rK39 RDT (20), compared to the *Leishmania* antigen ELISA and the rK39 ELISA used in this study. In Ethiopia, prevalence was found to be 12.8% in males, with being male and a concurrent malaria infection found to be risk factors for ALI (20). Furthermore, the population had relatively high median CD4 counts (377 cells/mm^3^ (IQR: 250-518)) with generally good overall ART adherence (20). In this study, median CD4 counts were 443 cells/mm^3^ (IQR: 303-595) with the majority of participants (75.2%) on ART for 12 months or more.  Much of the data on ALI in PLHIV has been collected in areas where *Leishmania infantum* is endemic (19,27,28). A study in Brazil used PCR, rK39 ELISA, indirect fluorescent antibody test, and an ELISA based on a crude *L. infantum* preparation, and found the prevalence of ALI in PLHIV to be 20.2% (27). More recently, a study in Brazil used the rK39 ELISA, rK39 RDT, DAT, KAtex, and PCR to estimate prevalence of ALI in PLHIV, and found prevalence to be 9.1% (28). Further, a meta-analysis of studies in PLHIV in *L. infantum* endemic areas found the prevalence of ALI to be 11.8% (19). Together these studies provide vital data to inform programmatic policy in a population at risk of poor disease outcomes. |
| Other information | |  | | |
| Funding | 22 | Give the source of funding and the role of the funders for the present study and, if applicable, for the original study on which the present article is based | 22 | This work was funded by Medecins Sans Frontiers, Spain, who fulfilled a sponsor-investigator role in the study. Additional funding in kind was provided by the The Medical Research Council (MRC) Doctoral Training Partnership (DTP) (MR/N013514/1). |

*Give information separately for cases and controls in case-control studies and, if applicable, for exposed and unexposed groups in cohort and cross-sectional studies.

**Note:** An Explanation and Elaboration article discusses each checklist item and gives methodological background and published examples of transparent reporting. The STROBE checklist is best used in conjunction with this article (freely available on the Web sites of PLoS Medicine at http://www.plosmedicine.org/, Annals of Internal Medicine at http://www.annals.org/, and Epidemiology at http://www.epidem.com/). Information on the STROBE Initiative is available at www.strobe-statement.org.
