# Supplementary material for: Prevalence and determinants of asymptomatic Leishmania infection in HIV-infected individuals living within visceral leishmaniasis endemic areas of Bihar, India
Source: PLoS Negl Trop Dis. 2022 Aug 30;16(8):e0010718. doi: 10.1371/journal.pntd.0010718 (PMC9467307; doi:10.1371/journal.pntd.0010718)
Supplement: S1 Table — (DOCX) [file pntd.0010718.s002.docx]

**S1 Table. Household related risk factors for asymptomatic *Leishmania* infection (ALI) in PLHIV including the *Leishmania* antigen ELISA in addition to qPCR, rK39 ELISA and RDT in the definition of ALI.**

|  | **All**  **N (%)** | | **Non- ALI**  **N (%)** | **ALI**  **N (%)** | **Odds Ratio (95% CI)** | **P value** |
| --- | --- | --- | --- | --- | --- | --- |
| Sex | | | | | | |
| Female | 694 (53.5) | 637 (54) | | 57 (49.1) | Ref |  |
| Male | 602 (46.5) | 543 (46) | | 59 (50.9) | 1.2 (0.8, 1.8) | 0.32 |
| Age | | | | | | |
| 18-29 | 174 (13.4) | 154 (13.1) | | 20 (17.2) | Ref |  |
| 30-44 | 731 (56.4) | 679 (57.5) | | 52 (44.8) | 0.6 (0.3, 1.02) | 0.06 |
| 45-59 | 329 (25.4) | 289 (24.5) | | 40 (34.5) | 1.1 (0.6, 1.9) | 0.83 |
| ≥ 60 | 62 (4.8) | 58 (4.9) | | 4 (3.4) | 0.5 (0.1, 1.7) | 0.33 |
| Household size | | | | | | |
| < 5 | 395 (30.5) | 371 (31.4) | | 24 (20.7) | Ref |  |
| ≥ 5 | 901 (69.5) | 809 (68.6) | | 92 (79.3) | 1.8 (1.1, 2.8) | **0.02** |
| Socioeconomic status | | | | | | |
| 1 or 2 | 130 (10.1) | 118 (10.0) | | 12 (10.3) | Ref |  |
| 3 | 333 (25.7) | 291 (24.7) | | 42 (36.2) | 1.4 (0.7, 2.8) | 0.31 |
| 4 | 513 (39.6) | 473 (40.2) | | 40 (34.5) | 0.8 (0.4, 1.6) | 0.59 |
| 5 | 318 (24.6) | 296 (25.1) | | 22 (19) | 0.7 (0.4, 1.5) | 0.4 |
| Type of house | | | | | | |
| Brick | 619 (47.8) | 560 (47.5) | | 59 (50.9) | Ref |  |
| Mud | 396 (30.6) | 363 (30.8) | | 33 (28.4) | 0.9 (0.6, 1.4) | 0.52 |
| Thatched | 281 (21.7) | 257 (21.8) | | 24 (20.7) | 0.9 (0.5, 1.5) | 0.63 |
| Proximity to pond | | | | | | |
| No | 1028 (79.3) | 929 (78.7) | | 99 (85.3) | Ref |  |
| Yes | 268 (20.7) | 251 (21.3) | | 17 (14.7) | 0.6 (0.4, 1.1) | 0.09 |
| Proximity to livestock | | | | | | |
| No | 534 (41.2) | 489 (41.4) | | 45 (38.8) | Ref |  |
| Yes | 762 (58.8) | 691 (58.6) | | 71 (61.2) | 1.1 (0.8, 1.7) | 0.58 |
| Time of last IRS (months) | | | | | | |
| Never | 144 (11.1) | 133 (11.3) | | 11 (9.5) | 0.8 (0.4, 1.5) | 0.41 |
| < 6 | 998 (77) | 900 (76.3) | | 98 (84.5) | Ref |  |
| 6-12 | 124 (9.6) | 118 (10) | | 6 (5.2) | 0.5 (0.2, 1.1) | 0.07 |
| > 12 | 30 (2.3) | 29 (2.5) | | 1 (0.9) | 0.3 (0.01, 2.0) | 0.35 |
| Number of IRS in last 18 months | | | | | | |
| 0 | 140 (10.8) | 129 (10.9) | | 11 (9.5) | Ref |  |
| 1 | 132 (10.2) | 127 (10.8) | | 5 (4.3) | 0.5 (0.2, 1.4) | 0.15 |
| 2 | 642 (49.5) | 588 (49.8) | | 54 (46.6) | 1.1 (0.6, 2.1) | 0.83 |
| > 2 | 382 (29.5) | 336 (28.5) | | 46 (39.7) | 1.6 (0.8, 3.2) | 0.17 |
| Contact with people with presumptive VL 50 metres around the house | | | | | | |
| No/Don’t know | 1236 (95.3) | 1125 (95.4) | | 111 (95.7) | Ref |  |
| Yes | 60 (4.6) | 55 (4.7) | | 5 (4.3) | 0.9 (0.3, 2.2) | 0.91 |
| Contact with people with presumptive PKDL 50 metres around the house | | | | | | |
| No/ Don’t know | 1278 (98.6) | 1164 (98.7) | | 114 (98.2) | Ref |  |
| Yes | 18 (1.4) | 16 (1.4) | | 2 (1.7) | 1.3 (0.2, 4.9) | 0.98 |
| Contact with people with cured VL/ PKDL 50 metres around the house | | | | | | |
| No/ Don’t know | 1262 (97.4) | 1151 (97.5) | | 111 (95.7) | Ref |  |
| Yes | 34 (2.6) | 29 (2.5) | | 5 (4.3) | 1.8 (0.6, 4.5) | 0.26 |
| Use bed nets while sleeping | | | | | | |
| Mostly  (>80%) | 1177 (90.8) | 1073 (90.9) | | 104 (89.7) | Ref |  |
| Never (0%) | 30 (2.3) | 27 (2.3) | | 3 (2.6) | 1.2 (0.2, 3.8) | 0.74 |
| Rarely  (1-49%) | 22 (1.7) | 18 (1.5) | | 4 (3.4) | 2.3 (0.6, 7.1) | 0.13 |
| Sometimes  (50- 80%) | 67 (5.2) | 62 (5.3) | | 5 (4.3) | 0.8 (0.3, 2.1) | 0.7 |
