# Supplementary material for: Prevalence and determinants of asymptomatic Leishmania infection in HIV-infected individuals living within visceral leishmaniasis endemic areas of Bihar, India
Source: PLoS Negl Trop Dis. 2022 Aug 30;16(8):e0010718. doi: 10.1371/journal.pntd.0010718 (PMC9467307; doi:10.1371/journal.pntd.0010718)
Supplement: S2 Table — (DOCX) [file pntd.0010718.s003.docx]

**S2 Table. HIV related risk factors for asymptomatic *Leishmania* infection (ALI) in PLHIV including the *Leishmania* antigen ELISA in addition to qPCR, rK39 ELISA and RDT in the definition of ALI.**

|  | **All  N (%)** | **Non- ALI**  **N (%)** | **ALI N (%)** | **Odds Ratio (95%CI)** | **P value** |
| --- | --- | --- | --- | --- | --- |
| Time on ART |  |  |  |  |  |
| ≥ 12 months | 974 (75.2) | 890 (75.4) | 84 (72.4) | Ref |  |
| 6-<12 months | 139 (10.7) | 123 (10.4) | 16 (13.8) | 1.4 (0.8, 2.4) | 0.27 |
| <6 months | 151 (11.7) | 140 (11.9) | 11 (9.5) | 0.8 (0.4, 1.6) | 0.60 |
| Pre-ART | 32 (2.5) | 27 (2.3) | 5 (4.3) | 2.0 (0.7, 5.0) | 0.2 |
| WHO clinical Stage |  |  |  |  |  |
| I | 1206 (93.1) | 1093 (92.6) | 113 (97.4) | Ref | 1 |
| II | 69 (5.3) | 68 (5.8) | 1 (0.9) | 0.1 (.004, 0.8) | **0.021** |
| III | 19 (1.5) | 17 (1.4) | 2 (1.7) | 1.1 (0.1, 4.9) | 1 |
| IV | 2 (0.2) | 2 (0.2) | 0 (0) | 0 (0, 51.8) | 1 |
| Anti-tubercular treatment (ATT) status |  |  |  |  |  |
| Not on ATT | 1144 (88.3) | 1038 (88) | 106 (91.4) | Ref |  |
| History of ATT | 134 (10.3) | 127 (10.8) | 7 (6) | 0.5 (0.3, 1.2) | 0.12 |
| Currently on ATT | 18 (1.4) | 15 (1.3) | 3 (2.6) | 2.0 (0.4, 7.1) | 0.24 |
| Time since HIV diagnosis (years) | | | | | |
| < 1 | 252 (19.4) | 225 (19.1) | 27 (23.3) | 1.3 (0.8, 2.1) | 0.25 |
| ≥ 1 | 1044 (80.6) | 955 (80.9) | 89 (76.7) | Ref |  |
| Body Mass Index (Kg/m^2^) |  |  |  |  |  |
| <16.5 | 123 (9.5) | 108 (9.2) | 15 (12.9) | 1.5 (0.8, 2.7) | 0.21 |
| 16.5-<18.5 | 292 (22.5) | 267 (22.6) | 25 (21.6) | 1 (0.6, 1.6) | 0.95 |
| 18.5-<25 | 761 (58.7) | 695 (58.9) | 66 (56.9) | Ref |  |
| ≥25 | 120 (9.3) | 110 (9.3) | 10 (8.6) | 1 (0.5, 1.9) | 0.9 |
| CD4 (cells / μL) |  |  |  |  |  |
| < 100 | 35 (2.7) | 29 (2.4) | 6 (5.2) | 2.3 (0.9, 5.8) | 0.06 |
| 100 - 199 | 104 (8.0) | 89 (7.5) | 15 (12.9) | 1.9 (1.1, 3.4) | **0.03** |
| 200 - 299 | 179 (13.8) | 164 (13.9) | 15 (12.9) | 1 (0.6, 1.8) | 0.93 |
| ≥ 300 | 978 (75.5) | 898 (76.1) | 80 (69) | Ref |  |
