# Supplementary material for: Prevalence and determinants of asymptomatic Leishmania infection in HIV-infected individuals living within visceral leishmaniasis endemic areas of Bihar, India
Source: PLoS Negl Trop Dis. 2022 Aug 30;16(8):e0010718. doi: 10.1371/journal.pntd.0010718 (PMC9467307; doi:10.1371/journal.pntd.0010718)
Supplement: S3 Table — (DOCX) [file pntd.0010718.s004.docx]

**S3 Table. Multivariable risk factor analysis for ALI** **in PLHIV including the *Leishmania* antigen ELISA in addition to qPCR, rK39 ELISA and RDT in the definition of ALI.**

| **Variable** | **aOR (95% CI)** | **P value** |
| --- | --- | --- |
| **Household size** |  |  |
| < 5 | **Ref** |  |
| ≥ 5 | 1.7 (1.1, 2.8) | **0.025** |
| **CD4 group** (cells / μL) |  |  |
| ≥ 300 | **Ref** |  |
| <100 | 2.6 (1, 6.9) | **0.05** |
| 100 - 199 | 2.0 (1.1, 3.7) | **0.032** |
| 200 - 299 | 1.0 (0.6, 1.8) | 0.933 |
| **Proximity to pond** | |  |
| No | **Ref** |  |
| Yes | 0.6 (0.3, 1.0) | **0.038** |
