# Supplementary material for: Prevalence and determinants of asymptomatic Leishmania infection in HIV-infected individuals living within visceral leishmaniasis endemic areas of Bihar, India
Source: PLoS Negl Trop Dis. 2022 Aug 30;16(8):e0010718. doi: 10.1371/journal.pntd.0010718 (PMC9467307; doi:10.1371/journal.pntd.0010718)
Supplement: S4 Table — (DOCX) [file pntd.0010718.s005.docx]

**S4 Table. Differences in baseline characteristics of individuals testing positive by urinary *Leishmania* antigen ELISA only in comparison other individuals.**

|  | **All (n=1296)** | **ALI**  **(n=96)** | **Non-ALI**  **(n=1200)** | **Urine positive (n=28)** | **Urine positive only (n=20)** |
| --- | --- | --- | --- | --- | --- |
| Median Age (IQR) | 39 (33-46) | 41 (33-50) | 39 (33-46) | 42 (34-46) | 42 (30-47) |
| Female - N (%) | 694 (53.5) | 46 (47.9) | 648 (54.0) | 15 (53.6) | 11 (55.0) |
| Median time in months on ART (IQR) | 33 (14-60) | 32 (12-63) | 33 (14-60) | 36 (14-59) | 37 (13-53) |
| Mean CD4 counts in cells/mm^3^ (SD) | 466 (230) | 400 (227) | 471 (229) | 442 (263) | 524.5 (245) |
